# The Contribution of a Whey Protein Film Incorporated with Green Tea Extract to Minimize the Lipid Oxidation of Salmon (*Salmo salar* L.)

**DOI:** 10.3390/foods8080327

**Published:** 2019-08-08

**Authors:** Frederico V. R. Castro, Mariana A. Andrade, Ana Sanches Silva, Maria Fátima Vaz, Fernanda Vilarinho

**Affiliations:** 1 Department of Food and Nutrition, National Institute of Health Doutor Ricardo Jorge, I.P., Av. Padre Cruz, 1649-016 Lisbon, Portugal; 2 Faculty of Sciences, University of Lisbon, Campo Grande, 1749-016 Lisbon, Portugal; 3 Faculty of Pharmacy, University of Coimbra, Pólo III - Pólo das Ciências da Saúde, Azinhaga de Santa Comba, 3000-354 Coimbra, Portugal; 4 National Institute for Agricultural and Veterinary Research (INIAV), I.P., Rua dos Lagidos, Lugar da Madalena, 4485-655 Vairão, Vila do Conde, Portugal; 5 Center for Study in Animal Science (CECA), ICETA, University of Porto, 4050-313 Porto, Portugal; 6 IDMEC, Instituto Superior Técnico, Departamento de Engenharia Mecânica, University of Lisbon, 1049-001 Lisbon, Portugal

**Keywords:** active packaging, edible films, whey protein, aromatic plants, rosemary, green tea, fresh salmon, lipid oxidation

## Abstract

Active packaging is becoming progressively more significant as a response to the dynamic changes in current consumer demand and market tendencies. Active packaging is projected to interact directly with the packaged food or with the headspace within the package with the aim of maintaining or extending product quality and shelf-life. Aiming for sustainability, the potential application as biodegradable films of whey protein concentrate (WPC) was evaluated. Aromatic plant’s extracts present high antioxidant properties, representing an alternative for synthetic food additives. The main objective of this study was to verify the effectiveness of an edible WPC film incorporated with a plant-based extract on retarding the lipid oxidation of fresh salmon. Green tea extract (GTE) was chosen to be incorporated into the active film. Fresh salmon was packaged with the control film (WPC) and with active film (WPC–GTE). The oxidation level of non-packaged samples and packaged samples were tested for different storage times. Four methods were applied to evaluate lipid oxidation state of fresh salmon: peroxide value, *p*-anisidine value, thiobarbituric acid reactive substances (TBARS) assay, and monitoring of hexanal. The results obtained in this study indicate that the whey protein active film was successfully produced, and it was effective in delaying lipid oxidation of fresh salmon samples until the 14th day of storage.

## 1. Introduction 

The demand for healthy, nutritious, safe and more “near-natural” products has increased as well as the search of new technologies for food production, food processing, and preservation. The consumer concerns related to food’s quality are reflected in awareness in nutritional composition, bioactive components, and safety issues.

Lipid oxidation and microbial growth are factors that are responsible for the loss of quality and reduction of food’s shelf life, contributing to deterioration of food products [1]. The oxidation process consists in the degradation of lipids and is responsible for changing, among others, the taste, texture, and nutritional value of foods, especially those with high-fat content. In addition, foods contaminated by fungi and bacteria have social and economic implications, not only by reducing the quality of foods but also by causing potential life-threatening diseases. Innovative technologies in the food packaging and food industries include the development of new materials to ensure the safety and quality of foods. In this sense, in recent decades, the development of biodegradable packaging has deserved great attention from researchers. Indeed, they have been dedicated to the study of new materials obtained from renewable and/or biodegradable sources, as an alternative to synthetic plastic packaging, in order to reduce the environmental impact of conventional packaging [2]. In this sense, viable edible films and coatings have been successfully produced from proteins, polysaccharides, and lipids, individually, or in combination among them. These films, while compostable and biodegradable, can also be consumed with the food product [3]. Whey protein-based films have shown good barrier and mechanical properties compared to the best synthetic polymer films available in the market [4], and, in addition, they can complement the nutritional value of the packaged foods [5]. However, these films show some restrictions concerning the moisture barrier properties and mechanical features, therefore, some plasticizers can be added to the whey protein (WP) (e.g. sorbitol or glycerol) to obtain a better resistance to moisture, as well as to avoid brittleness while increasing extensibility and flexibility [4,6,7].

The main feature of active packaging is the intentional interaction between food packaging and environment with the aim of extending food’s shelf-life, due to the improvement of the safety or sensorial properties, preserving or even improving the quality of the product. In this sense, the employment of edible films and coatings, as carriers of active compounds, shows potential to be used in the field of active food packaging [1]. Natural compounds such as essential oils or extracts from aromatic plants, fruits, and vegetables may be used as additives with antioxidant and antimicrobial properties without negative effects on human welfare [8]. However, their use as food preservatives is often limited due to their strong flavor. In order to avoid this problem, essential oils can be incorporated into edible films [9]. The combination of aromatic plants extracts, for example, can broaden their spectrum of action, due to the possibility of synergism, which allows more effective applications, and consequently even more promising results [10,11].

Sources of bioactive ingredients including several polyphenolic components with antioxidant and pro-oxidant properties are Green tea (*Camellia sinensis* L.), from the Theaceae family, and Rosemary (*Rosmarinus officinalis* L.) from the Lamiaceae family, [12,13]. These compounds may be commonly obtained in the form of essential oils and plant extracts. The most abundant compounds with antioxidant activity in green tea extract are epigallocatechin-3-gallate and epicatechin-3-gallate. Both compounds belong to the flavanol monomers known as catechins [13]. In the rosemary extract, carnosic acid, carnosol, and rosmarinic acid are the phenolic compounds which most contribute to its antioxidant capacity [14]. These two aromatic plant extracts have been applied, as a preservative agent or incorporated in food packaging, to different food matrices aiming to extend its shelf-life. These matrices include seafood [15], sardines [16], chicken nuggets [17], Pacific white shrimp [18], hake fillets [19], and smoked salmon [20].

In this study, the major goal was to development a WP-based edible active packaging, incorporated with two aromatic plant extracts to be applied to fresh salmon (*Salmo salar* L.). Salmon is a high-fat content food with a high value in the market. Rosemary and green tea extract antioxidant capacity was evaluated through the DPPH (2,2-difenil-1-picrilhidrazilo) radical scavenging method and β-carotene bleaching assay. Moreover, total phenolics content (TPC), and total flavonoids content (TFC) was determined. The effectiveness of the new active film against the lipid oxidation of fresh salmon was evaluated through the peroxide value, *p*-anisidine value, and thiobarbituric acid reactive substances (TBARS) assay, for several storage times (0, 5, 7, 10, 12, 14, and 17 days). 

## 2. Materials and Methods

### 2.1. Green Tea and Rosemary Extracts

To obtain an ethanolic rosemary extract (RE), dried rosemary leaves were acquired in a local store in Lisbon, Portugal, between January and April of 2018. The leaves were ground using a granulator mill Grindomix GM200 (Retsch, Haan, Germany) and were mixed with absolute ethanol, the American Chemical Society(ACS) reagent grade (Merck, Darmstadt, Germany) in a 1:10 ratio (*w*/*v*). Then, the solution was mixed using a compact stirrer Edmund Bühler GmbH model KS-15 (Hechingen, Germany), for 30 min at room temperature (23 °C ± 2 °C), protected from the light. After this, the mixture was centrifuged using an Eppendorf AG centrifuge (model 5804R, Hamburg, Germany) at 11.952 g for 15 min, at 15 °C. The supernatant was moved to a pear-shaped evaporation flask and, the ethanol was evaporated using a rotary evaporator Büchi model R-210 (Labortechnik, Switzerland) at 35 °C until dryness. With the help of a spatula, the extract was removed and kept until use under vacuum conditions, protected from the light at 5 °C. 

The GTE was acquired online in January 2018 from MyProtein (Batch code: W733443838). It was kept at 5 °C, protected from the light. Different concentrations of each extract were prepared to evaluate their antioxidant capacity.

### 2.2. Determination of the Antioxidant Capacity of Extracts

In order to determine which extract has the most powerful antioxidant capacity, two assays were used, the DPPH radical scavenging activity assay and the β-carotene bleaching test. One of the most used assays to determine the antioxidant capacity of food samples is the DPPH radical scavenging activity assay, which is based on the capacity of a sample to capture the DPPH free radical. The inhibition of the radical is monitored by the decrease of the sample’s absorbance (λ = 515–517 nm). It is a fast and easy to apply method, performed at room temperature, eliminating the risk of sample degradation by heat [21,22,23]. On the other hand, the β-carotene bleaching test measures the sample capacity to inhibit or delay the oxidation of an emulsion of linoleic acid and β-carotene at 50 °C. In similitude to the DPPH radical assay, it is fast and easy to apply method [24,25]. 

It is known that the Total Phenolics Content (TPC) and the total Flavonoids Content (TFC) of a sample do not directly evaluate their antioxidant capacity. However, they represent important methods to asset the antioxidant capacity of samples since the phenolics and the flavonoids are the main responsible for the antioxidant capacity of plants [20].

#### 2.2.1. DPPH Radical Scavenging Activity

The method used in this study was based on the method developed by Moure et al. (2001) [26]. A methanolic solution of 14.2 µg/mL of DPPH (Sigma-Aldrich; Madrid, Spain), was prepared. In this case, 50 µL of each sample was mixed with 2 mL of the methanolic DPPH solution and kept in the dark for 30 min, at room temperature. The absorbance of the samples was measured using an Evolution 300 UV–Vis spectrophotometer (Thermo Scientific™, Altrincham, England) against methanol (λ = 515 nm). The inhibition percent (IP%) of DPPH was calculated by Equation (1).

(1) $$IP=\frac{CA-SA}{CA}\times 100$$

in which, *CA* represents the control absorbance and the *SA* represents the sample absorbance. A calibration curve was drawn using a standard solution of Trolox (6-hydroxy-2,5,7,8-tetramethylchroman-2-carboxylic acid), a well-known powerful synthetic antioxidant. The results are shown in IP% and µg of Trolox equivalents per ml of extract (µg TE/mL).

Also, the EC_50_ (concentration of the sample that causes a 50% inhibition of the DPPH radical) of each extract was obtained. In order to do so, the concentration of each extract (mg/mL) *versus* IP (%) was plotted. Equation curves were obtained and subsequently EC_50_ was calculated [27].

#### 2.2.2. β-carotene Bleaching Assay

The method used in this study was developed by Miller (1971) [28]. A solution of 2 mg/mL of β-carotene (Sigma-Aldrich; Madrid, Spain) in chloroform, ACS reagent grade (Merck; Darmstadt, Germany) was prepared. To an amount of 1 mL of the β-carotene solution, 200 mg of Tween^®^ 40 and 20 mg of linoleic acid (Sigma-Aldrich; Madrid, Spain) were added. The chloroform was evaporated in a rotary evaporator at 40 °C. The emulsion was completed by adding 50 mL of ultra-pure water obtained from a Milli-Q™ system and vigorously shaken.

In 200 µL of each sample was added 5 mL of the β-carotene and linoleic acid emulsion. The solutions were kept at 50 °C for 2 h and then their absorbance was read in an Evolution 300 UV–Vis spectrophotometer (Thermo Scientific™, England) at 470 nm, against water. The absorbance of the control assays (with 200 µL of ethanol, 5 mL of the β-carotene and linoleic acid emulsion) was read before and after submitting the sample to 50 °C for 2 h. The Antioxidant Activity Coefficient (AAC) was calculated by Equation (2).

(2) $$AAC=\frac{AS-AC2}{AC0-AC2}\times 1000$$

in which, *AS* is the absorbance of the samples after 2 h of reaction, the *AC0* is the absorbance of the control assay at the initial time (*t* = 0 min), and the *AC2* is the absorbance of the control assay after 2 h. 

#### 2.2.3. Total Phenolics Content (TPC)

The determination of TPC was performed according to the method described by Erkan et al. (2008) [14]. For this method, an aqueous solution (1:10, *v*/*v*) of Folin-Cioucalteu reagent (Sigma-Aldrich; Madrid, Spain) and an aqueous solution (60 mg/mL) of sodium carbonate were prepared. To 1 ml of sample, an amount of 7.5 mL of the Folin-Cioucalteu solution was added, after which it was shaken. After 5 min, 7.5 mL of the sodium carbonate solution was added, and the mixture was again shaken and kept at room temperature in the dark, for 2 h. The absorbance of the samples was measured at 725 nm in an Evolution 300 UV–Vis spectrophotometer (Thermo Scientific™, England). A calibration curve of gallic acid (Sigma-Aldrich; Madrid, Spain) was drawn. The results are expressed in mg of Gallic Acid equivalents/g of extract (mg GAE/g of extract).

#### 2.2.4. Total Flavonoids Content

The method described by Yoo et al. (2008) [29] was used for the determination of the total flavonoids content. Briefly, to 1 mL of the sample a quantity of 5 mL of ultra-pure water (obtained by a Milli-Q™ system) was added. Then, 0.3 mL of an aqueous solution of sodium nitrite (5%, *w*/*v*) was joint and homogenized. After 5 min, 0.6 mL of an aqueous solution of aluminum chloride (10%, *w*/*v*) was put together and the solution was homogenized. After 6 min, 2 mL of an aqueous solution of sodium hydroxide (1 M) and 2.1 mL of ultra-pure water were incorporated, and the solution was homogenized. Then, the absorbance was measured using an Evolution 300 UV–Vis spectrophotometer (Thermo Scientific™, England) at 510 nm. A calibration curve of epicatechin was drawn. The results are expressed in mg Epicatechin Equivalents/g of extract (mg ECE/g of extract).

### 2.3. Development of the WP Active Film

WP concentrate (WPC) (80%) was acquired from Glanbia Nutritionals (USA) and glycerol from Sigma-Aldrich (Germany). In order to produce the active film, the method of film preparation was adapted from the method described by Bahram et al. (2014) [30].

To study the glycerol effect on the WPC film, two percentages of glycerol were tested in Teflon^®^ surfaces respectively: 5 and 8% (*w*/*v*).

The films were produced using a casting technique, described by Bahram et al. (2014) [30]. To a quantity of 12.77 g of WPC, an amount of 105.53 mL of ultra-pure water from a MilliQ™ system was added and the solution was homogenized using an Ultra-Turrax IKA^®^ DI 25 basic (Staufen, Germany) for 2–5 min, at 9500 rpm. The solution was then submitted to a temperature of 80 °C in a thermostatic bath Memmert GmbH WV 14 (Hechingen, Germany). After 30 min, the solution was rapidly cooled to room temperature with the help of iced water. Afterwards, glycerol was added, the solution was homogenized, and poured in Teflon^®^ round surfaces of 29.5 cm diameter. For the active film, GTE was added (1 or 2%, *w*/*w*), the solution was homogenized and poured in equal Teflon^®^ surfaces. The films were kept in separated rooms during the drying process (about 48 h) in order to avoid potential migration of volatile active compounds from the active films to the control films. After the drying process, the salmon samples were immediately packaged with the films.

### 2.4. Effectiveness of the WP Active Film

Salmon (*Salmo salar* L.) was chosen as a food model due to its high-fat content and high value on the market, in order to evaluate the effectiveness of the WP active film in delaying or inhibiting lipid oxidation.

#### 2.4.1. Sample Preparation

A whole salmon, produced in aquaculture, was purchased at a local store in Lisbon, Portugal. The salmon was split into 30–35 g slices and kept at −80 °C, wrapped up in tracing paper and protected from the light until use.

As the films were manufactured, the slices were packaged with the control and the active films under vacuum conditions, in order to maximize contact between the films and the fish surface. Afterwards, the samples were stored at 5 °C, protected from light for different durations, namely, 5, 7, 10, 14, and 17 days. The fat oxidation status was measured at the end of each storage time. In order to understand salmon´s natural oxidation process, slices were kept wrapped in tracing paper at 5 °C, protected from light, and the fat oxidation was measured at the end of 0, 3, 5, 7, 10, and 12 days. All samples were analyzed at the end of each storage time.

#### 2.4.2. Fat Extraction

For the determination of fatty acids profile, peroxide value, and *p*-anisidine value, it was necessary to extract the samples fatness. The method used was described by Bligh & Dyer (1959) [31]. To 30 g of ground sample, 150 mL of chloroform (Merck, Darmstadt, Germany), 300 mL of analytical grade methanol (Merck, Darmstadt, Germany), and 120 mL of ultrapure water were added. The samples were submitted to homogenization with Ultra-Turrax at 8000 rpm for 2 min and then magnetic stirring for 30 min. After this, 150 mL of chloroform and 150 mL of a 1.5% sodium sulphate solution (*w*/*v*) (Merck, Darmstadt, Germany) were joint, followed by magnetic stirring for 2 min and transferred to a pear-shaped separatory funnel. The lower phase was filtered with a Whatman No. 4 filter with 1 g of anhydrous sodium sulphate. Lastly, the chloroform was evaporated at 40 °C with a rotary evaporator. The fatness was stored at −80 °C.

#### 2.4.3. Fatty Acids Profile

To determine the fatty acids profile of salmon’s samples after their respective storage time, a gas chromatographic method with flame ionization detection (GC-FID) was used [32]. An Agilent 7890 GC (Santa Clara, CA, EUA) coupled with an FID detector at 275 °C and with an injector at 280 °C was used to perform all GC analysis. Fatty acid methyl esters (FAMEs) were separated in a Supelco 62009-03 analytical column (100 m × 250 µm × 0.2 µm), in split mode. The gas was helium at 15.6 mL/min. The oven temperature ramp was as follows: 100 °C at zero min; increase to 204 °C in 15 min; increase to 214 °C in 10 min; increase to 240 °C in 16 min. The lipid standard used for the determination of the mixture of FAMEs was Supelco^TM^ C37 FAME Mix (47885-U, Sigma-Aldrich, Madrid, Spain).

The extraction of fatness from each sample was carried out according to the procedure described in the previous sub-section. Afterward, to 0.2 g of fatness, 2.5 mL of *n*-hexane was added, and the mixture was homogenized. Then, 0.25 mL of a 2 M methanolic solution of potassium hydroxide (a transesterification reagent) was incorporated, and the mixture was shaken and kept at room temperature for 30 min. The upper phase of the solution was used for the determination of fatty acids. An amount of 1 µL was injected in the GC-FID.

#### 2.4.4. Peroxide Value

To determine the peroxide value (PV), the method described by the American Oil Chemists’ Society [33] was applied. In this sense, to 0.1 g of salmon’s fatness, 10 mL of isooctane (EMSURE^®^ grade ACS), 15 mL of acetic acid (VWR—BDH, PROLABO), and 1 mL of a saturated solution of potassium iodide were added. Then, the samples were homogenized and kept in the dark for 5 min. An amount of 3 mL of a 10 mg/mL amide solution (Sigma-Aldrich, Germany) and 75 mL of ultrapure water were then joint to the solution, giving to it a dark-purple color. For the titration, in which the solution turned white, a 0.01 N sodium thiosulfate solution (Merck; Darmstadt, Germany) was used. The control samples were composed only by reagents. The peroxide value (meqO_2_/kg of fat) was calculated by Equation (3).

(3) $$PV=\frac{\left(S-B\right)\times N\times 1000}{m}$$

in which *S* is the volume of the sodium thiosulfate solution in the titration, *B* is the volume of the sodium thiosulfate solution in the control test, *N* is the molar concentration of the sodium thiosulfate solution, and *m* is the salmon’s fat (g).

#### 2.4.5. *p*-Anisidine Value

The determination of *p*-anisidine value was carried out according to the British Standard method [34]. In this case, to 0.5 g of fat, a quantity of 25 mL of *n*-hexane (spectrophotometric grade, BDH Prolabo; Leuven, Belgium) was added. In a U-2000 Hitachi spectrophotometer, the samples’ absorbance was measured, at 350 nm, against *n*-hexane. In 1 mL of a 2.5 mg/mL acetic acid solution of *p*-anisidine (Sigma-Aldrich, Madrid, Spain) an amount of 5 mL of sample was added and kept in the dark for 10 min, at room temperature (23 °C). The control samples were composed of 5 mL of *n*-hexane and 1 mL of the *p*-anisidine solution. The absorbance of the samples was measured again, against the control samples. The *p*-anisidine value was calculated by Equation (4).

(4) $$AV=\frac{25\times \left(1.2Abs2-Abs1\right)}{m}$$

in which *Abs2* is the samples’ absorbance after 10 min of reaction time, *Abs1* is the samples’ absorbance at the beginning of the reaction, and *m* is the salmon’s fat (g). 

#### 2.4.6. TBARS Assay

This procedure was performed as described by D. Miller (1998) [35]. To 5 g of ground salmon, 50 mL of a 10% (*v*/*v*) orthophosphoric acid (analytical grade, Merck; Darmstadt, Germany) trichloroacetic acid solution (analytical grade, Merck; Darmstadt, Germany) was included. Then, the samples were homogenized using Ultra-Turrax at 8000 rpm, for 2 min, and filtrated with Whatman No. 4. For a quantity of 5 mL of the filtered solution, an amount of 5 mL of a 2.9 mg/mL (*w*/*v*) thiobarbituric acid aqueous solution was gathered and submitted to 100 °C for 40 min. Afterward, samples were cooled down with ice for 15 min and the absorbances were measured, at 530 nm, against the control sample. The control sample was composed of 5 mL of the thiobarbituric acid aqueous solution and 5 mL of ultrapure water.

#### 2.4.7. Statistical Analysis

Results were expressed as mean ± standard deviations of at least three replicates. Differences among samples were tested using T-Test. All statistical analyses were tested at 0.05 level of probability, using the SPSS^®^ computer software (SPSS Inc., Chicago, IL, USA).

## 3. Results and Discussion

### 3.1. Antioxidant Capacity

To assess and compare the antioxidant capacity of green tea and rosemary extracts, DPPH radical scavenging activity assay and β-carotene bleaching assay were carried out. Moreover, the determination of TPC and TFC was performed.

#### 3.1.1. DPPH Radical Scavenging Activity and β-carotene Bleaching Assays

**Figure 1:**
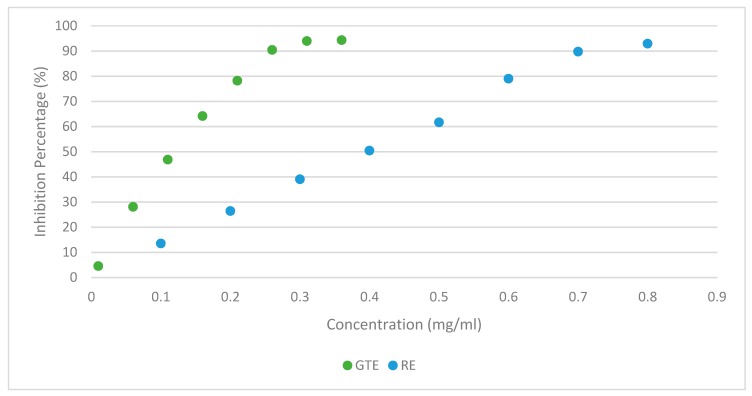
Study of Green Tea Extract (GTE) and Rosemary Extract (RE) antioxidant capacity through the DPPH assay inhibition percentage.

In the case of DPPH radical scavenging activity assay, Trolox was used as a standard. A calibration curve of Trolox was prepared, in the concentration range of 10–150 µg/mL (*y* = 0.6346*x* − 0.6143, *r*^2^ = 0.9975). Results of this assay are presented in inhibition percentage and in µg Trolox equivalents (TE) per mL. The DPPH inhibition assay results are shown in Figure 1 and Table 1.

In Figure 1, is represented the antioxidant capacity of the GTE and the RE at different concentrations. As can be observed, GTE reaches its full antioxidant potential between 0.25 and 0.3 mg/mL, which is very different from the RE that only reaches its full antioxidant potential between 0.7 and 0.8 mg/mL. It was observed that several studies expressed results of the DPPH assay in different units, which we are unable to compare with the present work. In order to perform a more accurate comparison of the antioxidant capacity, the EC_50_ (the necessary concentration of extract to capture 50% of DPPH free radicals in the solution) was also calculated. GTE presented 0.10 mg/mL, which was similar to the results obtained by Martins et al. (2018) [20] and Lorenzo et al. (2014) [36] of (0.12 mg/mL). As for rosemary extract, an EC_50_ of 0.43 mg/mL was calculated. Andrade et al. (2018) [37] and Pereira et al. (2017) [38], presented 0.38 and 0.13 mg/mL EC_50_ values, respectively, for rosemary ethanolic extracts. The EC_50_ values presented by Andrade et al. (2018) [37] are similar to the ones found in the present study. Pereira et al. (2017) [38], presented a lower EC_50_ value for rosemary extract but this was prepared using a different extraction procedure. In this case, dried samples of *R. officinalis* were extracted with 80% ethanol (*v*/*v*) at 70 °C in a water bath for 30 min. Then, the extract was filtered and the supernatant was lyophilized. This procedure allowed obtaining an extract with higher antioxidant capacity, which was similar to the one of GTE. Reis (2014) [39] has found that lyophilization allowed obtaining extracts with higher antioxidant capacity. In this work, the ethanolic rosemary extract obtained without lyophilization presented an EC_50_ of 0.37 mg/mL and the same lyophilized presented an EC_50_ of 0.24 mg/mL. Regarding the β-carotene bleaching assay results, GTE samples (1 mg/mL) and RE samples presented a similar antioxidant capacity coefficient (AAC = 379.24 ± 10.43 for GTE and AAC = 375.19 ± 0.98 for RE). Martins et al. (2018) [20] and Andrade et al. (2018) [37] registered higher results of AAC for GTE and RE, respectively, but they measure the AAC with a higher concentration (5 mg/mL).

#### 3.1.2. Determination of Total Phenolics and Flavonoids Content

In the determination of total phenolics content, there was a reaction of the Folin-Ciocalteau reagent with the active extracts, the oxidation of phenolic compounds and oxygen production occurred, which resulted in a blue-colored solution [40]. The content in phenolic compounds was calculated from the regression equation of the gallic acid calibration curve *y* = 7.0004*x* − 0.0775, with a coefficient of determination of 0.9993 in a range of 0.025−0.150 mg/mL, expressed in mg GAE/g extract, presented in Table 2. 

It was observed that GTE (0.1 mg/mL) presented a significant higher value of phenolic compounds content (443 ± 10.0 mg GAE/g extract) in comparison with RE for the same concentration (190.2 ± 2.97 mg GAE/g extract). In the literature, Kmiecik et al. (2018) [41], Lorenzo et al. (2014) [36], and Martins et al. (2018) [20] obtained values of 198, 391, and 416 mg GAE/g extract, respectively, for GTE samples. Rababah et al. (2004) [42] present the value of TFC in chlorogenic acid equivalents (CAE) in milligrams per gram of dry material which we are unable to compare with the present work. Comparing the results found in the literature with the results of this study, it can be concluded that the green tea and rosemary extracts analyzed in this study presented a higher value of phenolic compounds.

As far as flavonoids are concerned, these are a subclass of the phenolic compounds, which are potent metal chelators, and lipoperoxidation inhibitors [20]. They are plant’s secondary metabolites which have antioxidant activity in the presence of OH groups [43]. Epicatechin was used to obtain the calibration curve (*y* = 3.0021*x* + 0.0017) in a range of 0.025–0.175 mg/mL and a coefficient of determination of 0.9996. Results are expressed in mg ECE/g extract, presented in Table 2. RE samples presented a similar value of flavonoids content when compared with GTE, at 0.1 mg/mL. Martins et al. (2018) [20] presented results of 148 mg ECE/g extract in ethanolic green tea extract. The edaphoclimatic conditions can be a possible cause for the differences between the values of this study and the values mentioned above. 

Analyzing the antioxidant capacity assessment tests, it was verified that green tea extract had a stronger antioxidant power than the rosemary extract. Also, an inferior EC_50_ value of green tea was observed, which confirms the antioxidant power of the GTE. With these results as support, only the GTE was incorporated into the whey protein-based active films. 

### 3.2. Active Film Development 

The active films were composed of Whey Protein Concentrate (WPC), glycerol, water, and GTE. In order to assure the effectiveness of the films, it was necessary to optimize the percentage of glycerol and GTE incorporated.

#### Glycerol and Green Tea Extract

The plasticizer that was chosen was the glycerol because of its hydrophilic nature and low molecular weight, which make possible its incorporation in protein matrices. Also, glycerol increases resistance, flexibility, and extensibility in films [44]. 

Different combinations of glycerol and GTE were selected and tested based on the results obtained by Andrade et al. (2018) [37]. Therefore, 4 different films from F_1_ to F_4_ were tested, as depicted in Table 3.

F_1_ and F_2_ presented a fragile and brittle consistency, while F_3_ was pasty. Lastly, F_4_ was a homogeneous, resistant, and flexible film, without structure deterioration at the removal from the Teflon surface. Therefore, the percentages of F_4_ (1% GTE and 8% glycerol) were chosen to produce the active film.

### 3.3. Lipid Oxidation Assessment

#### 3.3.1. Determination of Fatty Acids

Table 4 shows the nutritional composition of fresh salmon from two different sources and it is possible to note a great difference in the fat content between wild and farmed salmon. The determination of lipids and assessment of fatty acids composition is of great importance for human health, as lipids are one of the three major constituents of food. The most used technique to identify fatty acids is gas chromatography with flame ionization detection [45,46].

The values of the fatty acids present were determined in fresh salmon with 0, 5, 7, and 10 days of storage at refrigerated temperature, in salmon that was packed with the control film for 5 and 7 days, respectively, and in the salmon that was packed with the active film for 5 and 7 days, respectively.

In Table 5 the value of saturated fatty acids results from the sum Ʃ (C10:0 + C12:0 + C14:0 + C15:0 + C16:0 + C17:0 + C18:0 + C20:0 + C21:0 + C24:0), the value of monounsaturated fatty acids results from the sum Ʃ (C14:1 + C16:1c + C17:1c + C18:1 + C20:1 + C24:1) and the value of polyunsaturated fatty acids results from the sum Ʃ (C18:2c(*n*6) + C18:3c(*n*3) + C18:3c(*n*6) + C20:4(*n*6) + C22:2 + C22:5(n3) + C22:6(*n*3)).

As we can verify in Table 5 the values of fatty acids present in fresh salmon are according to the literature consulted [47]. 

Analyzing the fresh fatty acid values of salmon over time, with salmon values when packed with control film and with active film, in the same storage period, 5 and 7 days respectively, we can verify that there are no significant changes, leading us to suppose that there was no degradation of the fatty acids throughout these storage times.

This can be explained by the short storage time at which the samples were subjected. Probably results from the period of 30 days of storage would show signs of degradation of the fatty acids.

#### 3.3.2. Peroxide Value

Peroxide serves as a useful index of the extent of oxidation of lipids, fats, and oils. The peroxide value shows the degree of peroxidation and measures the amount of total peroxides in the substance. The radical species formed in the peroxidation process degrade fatty acids and other components of the lipids [49,50]. 

Regarding the results, peroxide was not detected for all storage times and samples, with the exception of the non-packaged salmon 5th (3.28 meq O_2_/kg) and the 10th (1.63 meq O_2_/kg) day of storage.

After the 5th day of storage time, the peroxide value decrease. This fact may be due to the higher deterioration rate of hydroperoxides over its production (the development of lipid oxidation is the decomposition of peroxides in secondary products) or may be due to its reaction with other compounds [51].

As in the present work, Martins et al. (2018) [20] also show that there was no progression of peroxide values along storage time in salmon samples. They studied smoked salmon packaged in the active film with green tea incorporated, and the highest peroxide level registered was 5 meq O_2_/kg, observed after 30 days of storage time.

#### 3.3.3. Determination of *p*-anisidine Value

The *p*-anisidine value (AV) method measures the secondary oxidation compounds, primarily 2-alkenals and 2,4-alkadienals produced due to hydroperoxide decomposition, and it is more sensitive to unsaturated aldehydes [52]. The *p*-anisidine reacts with aldehydes, leading to the formation of yellow compounds [53].

**Figure 2:**
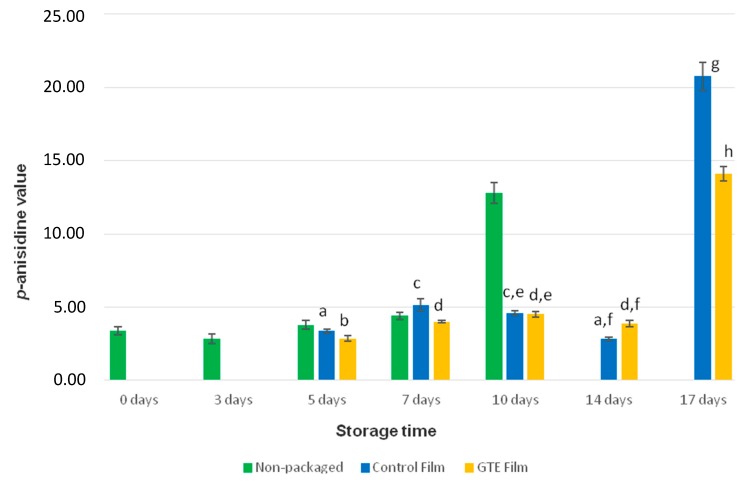
Results of *p*-anisidine value determination of fresh salmon samples packaged with the control film (without GTE), packaged with the active film (with GTE) and non-packaged salmon. Different letters are for significant differences (*p* < 0.05).

In Figure 2, it was verified that only for the 14th day (3.86 ± 0.09 *p*-anisidine value) the samples packaged with the control film presented lower levels of *p*-anisidine than the samples packaged with the active film. In all other storage times, the opposite was observed. However, the differences found at this storage time were not statistically different (*p* > 0.05). The longest storage period studied for non-packaged samples was 12 days of storage time, to which corresponds the highest *p*-anisidine value (14.20 ± 1.11). 

After 17 days of storage, the *p*-anisidine value differences were significantly different between fish samples packaged with control and active films, so these results allow concluding that the active film is effective, at this storage period, because it has minimized lipid oxidation of salmon. These results are in accordance with those of Martins et al. (2018) [20], for which, after 60 days in the dark, at 5 °C, the smoked salmon samples packaged with an active polylactic acid-base film with GTE, presented lower values of *p*-anisidine than the ones packaged with the control film (without GTE).

#### 3.3.4. TBARS Assay

**Figure 3:**
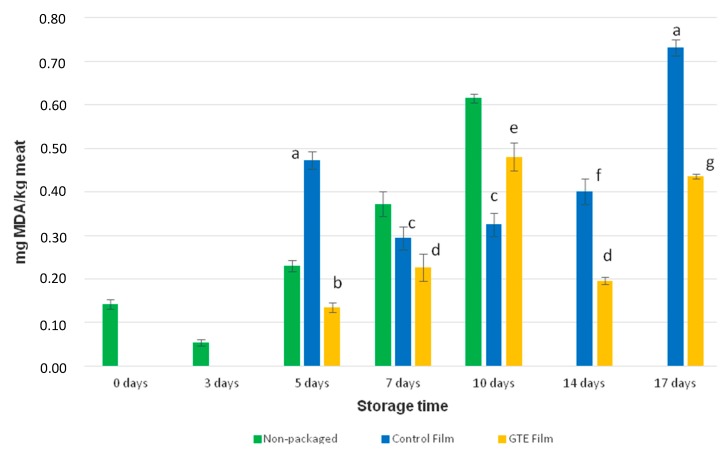
Results of TBARS assay for fresh salmon samples packaged with the control film (without GTE), packaged with the active film (with GTE) and non-packaged salmon. The red line stands for the threshold of off-odor perception by consumers. MDA—Malonaldehyde. Different letters are for significant differences (*p* < 0.05).

TBARS is probably the oldest and one of the most widely used assays for measuring lipid peroxidation end product malondialdehyde (MDA), which is a reactive aldehyde produced by lipid peroxidation of polyunsaturated fatty acids [54]. The MDA is the main aldehyde produced during unsaturated fatty acids decomposition to hydroperoxides, in primary oxidation [35,55]. According to a study by Remya et al. (2016) [56], consumers settled that the off-odor perception of microbial growth corresponds to 0.5 mg MDA/kg sample, in barracuda fish. Analyzing the Figure 3, the non-packaged fresh salmon at 10 days of storage time showed a value higher than 0.5 mg MDA/kg sample. The highest result of MDA for non-packaged fresh salmon was found at 12 days (0.7 ± 0.02 mg MDA/kg sample). 

Regarding to packaged samples, except for the 10th day, the samples packaged with the active film presented lower levels of MDA than the samples packaged with the control film. According to Figure 3 even at the longest storage period studied (17 days), the samples packaged with the active films still presented values of MDA lower than 0.5 mg MDA/kg sample, indicating that this active packaging protects fresh salmon against lipid oxidation. To investigate the effectiveness of a polylactic acid-base film with green tea extract incorporated in the lipid oxidation of smoked salmon, Martins et al. (2018) [20] kept the samples in the dark at 5 °C, for up to 60 days. The TBARS assay was applied to evaluate lipid oxidation. According to their results, samples packaged with the active film presented lower values of MDA than the ones packaged with the control film. Thus, it was acceptable to conclude that the active film was effective in delaying lipid oxidation in smoked salmon. In turn, Özen & Soyer (2018) [57] concluded that GTE in the concentration of 100 mg extract of kg sample delays the process of lipid oxidation in mackerel, once TBARS assay showed an increase in MDA over time in all samples. However, the comparison of samples subjected to GTE with the control samples demonstrated lower levels of MDA in the first ones. In this research work [56] the samples were stored for 6 months at −18 °C, with monthly analysis.

## 4. Conclusions

The green tea extract presented the higher antioxidant capacity evaluated by DPPH radical scavenging activity and β-carotene bleaching assays, in comparison with rosemary extract. Moreover, GTE also showed to have higher total phenolics content. Consequently, it was chosen to be incorporated in a whey protein-based film. The process of manufacture of the active film was optimized. Different concentrations of glycerol (plasticizer) and the active extract were tested. The percentages that permit to obtain the best results were 8% (*w*/*w*) of glycerol and 1% (*w*/*w*) of green tea extract. The active whey protein-based film was evaluated for its efficacy in decreasing the lipid oxidation status of high-fat food, fresh salmon. *p*-Anisidine value and the TBARS tests allowed to conclude that the active film (with green tea extract) was effective in delaying the lipid oxidation of salmon samples fresh compared to the control film (without green tea extract). The new active film can be applied to separate slices of salmon or to wrap pieces of salmon. The advantage of this primary packaging is the fact that it can also be consumed with the food product. However, further work is needed to evaluate the mechanical properties of the active films vs. control films and microbiological evaluation of the salmon samples. For potential commercialization, a sensory analysis through a panel of tasters will be necessary to evaluate possible changes in the organoleptic characteristics of the salmon samples for their acceptability. 

## Figures and Tables

**Table 1:** Results of DPPH radical assay expressed in Inhibition Percentage (%) and µg Trolox equivalents per ml (µg TE/mL) (mean ± Standard Deviation (SD)) for Green Tea Extract (GTE) and Rosemary Extract (RE). Different letters are for significant differences (*p* < 0.05).

Concentration (mg/mL)	DPPH Assay			
	Inhibition Percentage (%)		μg TE/mL	
	GTE	RE	GTE	RE
0.1	51.43 ± 1.98 ^a^	13.36 ± 0.24 ^b^	83.74 ± 3.73 ^A^	22.02 ± 0.37 ^B^
0.2	86.62 ± 0.12 ^c^	24.43 ± 0.38 ^d^	133.41 ± 7.03 ^C^	39.47 ± 0.59 ^D^

GTE—Green Tea Extract; RE—Rosemary Extract; TE—Trolox Equivalents; SD—Standard deviation.

**Table 2:** Results of total phenolic compounds (expressed in mg Gallic Acid equivalents per g extract (mg GAE/g extract)) and total flavonoids results, (expressed in mg Epicatechin equivalents per g extract (mg ECE/g extract)), mean ± SD. Different letters are for significant differences (*p* < 0.05).

Phenolic Content (mg GAE/g Extract)		Flavonoid Content (mg ECE/g Extract)	
GTE	RE	GTE	RE
443.55 ± 10.00 ^a^	190.23 ± 2.97 ^b^	119.81 ± 11.70 ^A^	118.69 ± 5.09 ^B^

GAE—Gallic Acid Equivalents; ECE—Epicatechin Equivalents; GTE—Green Tea Extracts; RE—Rosemary Extract.

**Table 3:** Films tested for the optimization of glycerol and green tea extract.

Films	GTE (%, *w*/*w*)	Glycerol (%, *w*/*w*)
F_1_	2	5
F_2_	1	5
F_3_	2	8
F_4_	1	8

GTE—Green Tea Extract.

**Table 4:** Fresh salmon (*Salmon salar* L.) nutritional composition according to the Portuguese Food Composition Table [47] and the United States Department of Agriculture (USDA, 2018) [48] Values per 100 g.

Nutrient	Unit	Wild Salmon	Farmed Salmon	Salmon (*Salmon salar* L.)
Water	g	68.5	65.9	60.5
Energy	kcal	142	208	262
Protein	g	19.8	20.4	16.2
Total lipid	g	**6.3**	**13.4**	**21.9**
Ash	g	2.5	1.1	1.3
Carbohydrate (by difference)	g	0	0	0
Fiber (total dietary)	g	0	0	0
**Minerals**				
Calcium (Ca)	mg	12	9	12
Iron (Fe)	mg	0.8	0.3	0.5
Magnesium (Mg)	mg	29	27	23
Phosphorus (P)	mg	200	240	210
Potassium (K)	mg	490	363	300
Sodium (Na)	mg	44	59	38
Zinc (Zn)	mg	0.6	0.4	0.5
Copper (Cu)	mg	0.3	0.05	-
Manganese (Mn)	mg	0.02	0.01	-
Selenium (Se)	μg	36.5	24	-
**Vitamins**				
Thiamin	mg	0.2	0.2	0.18
Riboflavin	mg	0.4	0.2	0.04
Niacin	mg	7.9	8.7	3.6
Pantothenic acid	mg	1.7	1.5	-
Vitamin B-6	mg	0.8	0.6	0.45
Folate (DFE)	μg	25	26	10
Vitamin B-12	μg	3.2	3.2	1.9
Vitamin A (RAE)	μg	12	58	33
Retinol	μg	12	58	-
Vitamin A (IU)	IU	40	193	-
**Lipids**				
Fatty acids (total saturated)	g	1.0	3.1	4.2
14:0	g	0.1	0.6	-
16:0	g	0.6	1.9	-
18:0	g	0.2	0.5	-
Fatty acids (total monounsaturated)	g	2.1	3.8	10
16:1 undifferentiated	g	0.3	0.8	-
18:1 undifferentiated	g	1.4	2.7	-
20:1	g	0.2	0.3	-
22:1 undifferentiated	g	0.3	0	-
Fatty acids (total polyunsaturated)	g	2.5	3.9	5.1
18:2 undifferentiated	g	0.2	0.9	-
18:3 undifferentiated	g	0.3	0.2	-
18:4	g	0.1	0.1	-
20:4 undifferentiated	g	0.3	0.1	-
20:5 *n*-3 (EPA)	g	0.3	0.9	-
22:5 *n*-3 (DPA)	g	0.3	0.4	-
22:6 *n*-3 (DHA)	g	1.1	1.1	-
Cholesterol	mg	55	55	40
Reference	-	USDA	USDA	TCAP

DFE—Dietary Folate Equivalent; RAE—Retinol Activity Equivalent; IU—International Unit; EPA—Eicosapentaenoic Acid; DPA—Docosapentaenoic Acid; DHA—Docosahexaenoic Acid.

**Table 5:** Fatty acids present in fresh salmon, non-packaged, packaged with control film and packaged with active film.

Fatty Acids	Percentage of Fatty Acids in g/100 g of Sample							
	Non-packaged Fresh Salmon				Salmon Packed with Control Film		Salmon Packed with Active Film	
	0 Days	5 Days	7 Days	12 Days	5 Days	7 Days	5 Days	7 Days
Saturated	3.7	3.7	3.6	3.6	3.6	3.6	3.6	3.6
Monounsaturated	9.7	9.5	9.7	9.7	9.7	9.8	9.6	9.7
Polyunsaturated	7.5	7.6	7.6	7.6	7.6	7.5	7.6	7.6
Polyunsaturated of which *n*3	1.3	1.7	1.4	1.4	1.5	1.3	1.5	1.5
Polyunsaturated of which *n*6	6.0	5.8	6.0	6.0	5.9	5.9	5.9	5.9
trans	0.05	0.04	0.04	0.05	0.05	0.05	0.05	0.05

## References

[1] Ribeiro dos Santos R. (2016). Development of protein active film incorporated with combination of essential oils: characterization and effectiveness. Ph.D. Thesis.

[2] Khwaldia K., Perez C., Banon S., Desobry S., Hardy J. (2004). Milk Proteins for Edible Films and Coatings. Crit. Rev. Food Sci. Nutr..

[3] Tomasula P.M. (2009). Using dairy ingredients to produce edible films and biodegradable packaging materials. Dairy-Derived Ingredients.

[4] Ramos Ó.L., Fernandes J.C., Silva S.I., Pintado M.E., Malcata F.X. (2012). Edible Films and Coatings from Whey Proteins: A Review on Formulation, and on Mechanical and Bioactive Properties. Crit. Rev. Food Sci. Nutr..

[5] Oliveira S.P.L.F., Bertan L.C., De Rensis C.M.V.B., Bilck A.P., Vianna P.C.B. (2017). Whey protein-based films incorporated with oregano essential oil. Polímeros.

[6] Hong-jiang W., Cheng S., Li-qiang H. Preparation and Properties of Whey Protein Packaging Film. Proceedings of the 17th IAPRI World Conference on Packaging.

[7] Ribeiro-Santos R., Motta J.F.G., Melo N.R., Costa B.S., Gonçalves S.M. (2018). Elaboration of active films with whey protein isolate and concentrate. Int. Food Res. J..

[8] Marturano V., Bizzarro V., Ambrogi V., Cutignano A., Tommonaro G., Abbamondi G.R., Giamberini M., Tylkowski B., Carfagna C., Cerruti P. (2019). Light-Responsive Nanocapsule-Coated Polymer Films for Antimicrobial Active Packaging. Polymers.

[9] Atarés L., Chiralt A. (2016). Essential oils as additives in biodegradable films and coatings for active food packaging. Trends Food Sci. Technol..

[10] Cardoso L.G., Pereira Santos J.C., Camilloto G.P., Miranda A.L., Druzian J.I., Guimarães A.G. (2017). Development of active films poly (butylene adipate co-terephthalate)—PBAT incorporated with oregano essential oil and application in fish fillet preservation. Ind. Crops Prod..

[11] De Moraes Crizel T., Haas Costa T.M., de Oliveira Rios A., Hickmann Flôres S. (2016). Valorization of food-grade industrial waste in the obtaining active biodegradable films for packaging. Ind. Crops Prod..

[12] European Food Safety Authority (2008). Use of rosemary extracts as a food additive—Scientific Opinion of the Panel on Food Additives, Flavourings, Processing Aids and Materials in Contact with Food. EFSA J..

[13] Kim H.-S., Quon M.J., Kim J. (2014). New insights into the mechanisms of polyphenols beyond antioxidant properties; lessons from the green tea polyphenol, epigallocatechin 3-gallate. Redox Biol..

[14] Erkan N., Ayranci G., Ayranci E. (2008). Antioxidant activities of rosemary (*Rosmarinus Officinalis* L.) extract, blackseed (*Nigella sativa* L.) essential oil, carnosic acid, rosmarinic acid and sesamol. Food Chem..

[15] Dehghani S., Hosseini S.V., Regenstein J.M. (2018). Edible films and coatings in seafood preservation: A review. Food Chem..

[16] López-de-Dicastillo C., Gómez-Estaca J., Catalá R., Gavara R., Hernández-Muñoz P. (2012). Active antioxidant packaging films: Development and effect on lipid stability of brined sardines. Food Chem..

[17] Kristam P., Eswarapragada N.M., Bandi E.R., Tumati S.R. (2016). Evaluation of edible polymer coatings enriched with green tea extract on quality of chicken nuggets. Vet. World.

[18] Dong Z., Xu F., Ahmed I., Li Z., Lin H. (2018). Characterization and preservation performance of active polyethylene films containing rosemary and cinnamon essential oils for Pacific white shrimp packaging. Food Control..

[19] López de Lacey A.M., López-Caballero M.E., Montero P. (2014). Agar films containing green tea extract and probiotic bacteria for extending fish shelf-life. LWT-Food Sci. Technol..

[20] Martins C., Vilarinho F., Sanches Silva A., Andrade M., Machado A.V., Castilho M.C., Sá A., Cunha A., Vaz M.F., Ramos F. (2018). Active polylactic acid film incorporated with green tea extract: Development, characterization and effectiveness. Ind. Crops Prod..

[21] Brand-Williams W., Cuvelier M.E., Berset C. (1995). Use of a free radical method to evaluate antioxidant activity. LWT-Food Sci. Technol..

[22] Bondet V., Brand-Williams W., Berset C. (1997). Kinetics and Mechanisms of Antioxidant Activity using the DPPH· Free Radical Method. LWT-Food Sci. Technol..

[23] Sánchez-Moreno C., Larrauri J.A., Saura-Calixto F. (1998). A procedure to measure the antiradical efficiency of polyphenols. J. Sci. Food Agric..

[24] Kato S., Aoshima H., Saitoh Y., Miwa N. (2009). Highly hydroxylated or γ-cyclodextrin-bicapped water-soluble derivative of fullerene: The antioxidant ability assessed by electron spin resonance method and β-carotene bleaching assay. Bioorg. Med. Chem. Lett..

[25] Phan-Thi H., Durand P., Prost M., Prost E., Waché Y. (2016). Effect of heat-processing on the antioxidant and prooxidant activities of β-carotene from natural and synthetic origins on red blood cells. Food Chem..

[26] Moure A., Franco D., Sineiro J., Domínguez H., Núñez M.J., Lema J.M. (2001). Antioxidant activity of extracts from Gevuina avellana and Rosa rubiginosa defatted seeds. Food Res. Int..

[27] Cruz J.M., Conde E., Domínguez H., Parajó J.C. (2007). Thermal stability of antioxidants obtained from wood and industrial wastes. Food Chem..

[28] Miller H.E. (1971). A simplified method for the evaluation of antioxidants. J. Am. Oil Chem. Soc..

[29] Yoo K.M., Lee C.H., Lee H., Moon B., Lee C.Y. (2008). Relative antioxidant and cytoprotective activities of common herbs. Food Chem..

[30] Bahram S., Rezaei M., Soltani M., Kamali A., Ojagh S.M., Abdollahi M. (2014). Whey Protein Concentrate Edible Film Activated with Cinnamon Essential Oil. J. Food Process. Preserv..

[31] Bligh E.G., Dyer W.J. (1959). A rapid method of total lipid extraction and purification. Can. J. Biochem. Physiol..

[32] ISO 5509:2000 (2000). Animal and Vegetable Fats and Oils—Preparation of Methyl Esters of Fatty Acids. https://www.iso.org/standard/11560.html.

[33] The American Oil Chemists’ Society (1989). AOCS Official Method Cd 8b-90. Peroxide Value, Acetic Acid, Isooctane Method.

[34] (1998). BS 684-2.24 Methods of Analysis of Fats and Fatty Oils. Other methods. Determination of Anisidine Value.

[35] Miller D. (1998). Food Chemistry: A Laboratory Manual.

[36] Lorenzo J.M., Sineiro J., Amado I.R., Franco D. (2014). Influence of natural extracts on the shelf life of modified atmosphere-packaged pork patties. Meat Sci..

[37] Andrade M.A., Ribeiro-Santos R., Costa Bonito M.C., Saraiva M., Sanches-Silva A. (2018). Characterization of rosemary and thyme extracts for incorporation into a whey protein based film. LWT.

[38] Pereira D., Pinheiro R.S., Heldt L.F.S., de Moura C., Bianchin M., Almeida J.d.F., dos Reis A.S., Ribeiro I.S., Haminiuk C.W.I., Carpes S.T. (2017). Rosemary as natural antioxidant to prevent oxidation in chicken burgers. Food Sci. Technol..

[39] Reis A.R.d.S. (2014). Estudo de compostos bioactivos e vitaminas de plantas aromáticas e sua aplicação em embalagens alimentares activas. MSc Thesis.

[40] Rivelli D.P. (2011). Biodisponibilidade, distribuição tecidual e atividade antioxidante do extrato hidroetanólico de *Ilex paraguariensis* hidrolisado e não hidrolisado. Ph.D. Thesis.

[41] Kmiecik D., Gramza-Michałowska A., Korczak J. (2018). Anti-polymerization activity of tea and fruits extracts during rapeseed oil heating. Food Chem..

[42] Rababah T.M., Hettiarachchy N.S., Horax R. (2004). Total Phenolics and Antioxidant Activities of Fenugreek, Green Tea, Black Tea, Grape Seed, Ginger, Rosemary, Gotu Kola, and Ginkgo Extracts, Vitamin E, and tert-Butylhydroquinone. J. Agric. Food Chem..

[43] Gutiérrez T.J., Ponce A.G., Alvarez V.A. (2017). Nano-clays from natural and modified montmorillonite with and without added blueberry extract for active and intelligent food nanopackaging materials. Mater. Chem. Phys..

[44] Kowalczyk D., Baraniak B. (2011). Effects of plasticizers, pH and heating of film-forming solution on the properties of pea protein isolate films. J. Food Eng..

[45] Cascant M.M., Breil C., Fabiano-Tixier A.S., Chemat F., Garrigues S., de la Guardia M. (2018). Determination of fatty acids and lipid classes in salmon oil by near infrared spectroscopy. Food Chem..

[46] Petrović M., Kezić N., Bolanča V. (2010). Optimization of the GC method for routine analysis of the fatty acid profile in several food samples. Food Chem..

[47] National Institute of Health Dr. Ricardo Jorge (INSA) Tabela da Composição de Alimentos. http://portfir.insa.pt/foodcomp/food?434.

[48] USDA Food Composition Databases. https://ndb.nal.usda.gov/ndb/.

[49] Mehta B.M., Darji V.B., Aparnathi K.D. (2015). Comparison of five analytical methods for the determination of peroxide value in oxidized ghee. Food Chem..

[50] Dermiş S., Can S., Doğru B. (2012). Determination of Peroxide Values of Some Fixed Oils by Using the mFOX Method. Spectrosc. Lett..

[51] Bou R., Codony R., Tres A., Decker E.A., Guardiola F. (2008). Determination of hydroperoxides in foods and biological samples by the ferrous oxidation—xylenol orange method: A review of the factors that influence the method’s performance. Anal. Biochem..

[52] Yang X., Boyle R.A. (2016). Sensory Evaluation of Oils/Fats and Oil/Fat–Based Foods. Oxidative Stability and Shelf Life of Foods Containing Oils and Fats.

[53] Guo Q., Gao S., Sun Y., Gao Y., Wang X., Zhang Z. (2016). Antioxidant efficacy of rosemary ethanol extract in palm oil during frying and accelerated storage. Ind. Crops Prod..

[54] Dasgupta A., Klein K., Dasgupta A., Klein K. (2014). Methods for Measuring Oxidative Stress in the Laboratory. Antioxidants in Food, Vitamins and Supplements.

[55] Osawa C.C., De Felício P.E., Gonçalves L.A.G. (2005). Teste de TBA aplicado a carnes e derivados: Métodos tradicionais, modificados e alternativos. Quim. Nova.

[56] Remya S., Mohan C.O., Bindu J., Sivaraman G.K., Venkateshwarlu G., Ravishankar C.N. (2016). Effect of chitosan based active packaging film on the keeping quality of chilled stored barracuda fish. J. Food Sci. Technol..

[57] Özalp Özen B., Soyer A. (2018). Effect of plant extracts on lipid and protein oxidation of mackerel (*Scomber scombrus*) mince during frozen storage. J. Food Sci. Technol..

